# Gene–Environment Interactions of Apoptosis-Related Polymorphisms and Urinary Polycyclic Aromatic Hydrocarbon (PAH) Metabolites in Relation to Sperm Cell Apoptosis Among Men Attending Infertility Clinics

**DOI:** 10.3390/toxics14010050

**Published:** 2025-12-31

**Authors:** Shiting Yi, Sitong Lin, Jiabin Xie, Zhihong Yang, Junxia You, Ximei Zhong, Hui Yang, Haiqing Lin, Qian Wang, Yajie Gong, Pan Yang, Yan Bai, Yingjun Chen

**Affiliations:** 1 First School of Clinical Medicine, The First Affiliated Hospital of Guangdong Pharmaceutical University, 19 Nonglinxia Road, Yuexiu District, Guangzhou 510080, China; 2 School of Public Health, Guangdong Pharmaceutical University, Guangzhou 510006, China; 3 Songgang People’s Hospital, 118 Longjing 2nd Road, Bao’an District, Shenzhen 518101, China; 4 Department of Public Health and Preventive Medicine, School of Medicine, Jinan University, Guangzhou 510006, China

**Keywords:** polycyclic aromatic hydrocarbons, sperm apoptosis, apoptosis-related gene, single nucleotide polymorphisms, gene-environment interaction

## Abstract

Polycyclic aromatic hydrocarbons (PAHs) are ubiquitous environmental endocrine disruptors (EDCs) that enter the human body through respiratory, digestive, and dermal exposure. Prolonged exposure has been associated with adverse health outcomes, including carcinogenicity, mutagenicity, and reproductive toxicity. However, whether genetic variation in apoptosis-related pathways modifies the reproductive effects of PAH exposure remains unclear. To investigate gene-environment interactions between urinary PAH metabolites and polymorphisms in apoptosis-related genes in relation to sperm apoptosis, we conducted a cross-sectional study involving 176 male participants from an infertility clinic in Wuhan, China, who completed structured questionnaires and provided biological samples. Ten OH-PAH metabolites in repeated urine samples were measured, along with genotyping of single-nucleotide polymorphisms (SNPs) at apoptosis-related genes (Fas, FasL, and caspase-3) in whole blood DNA, and sperm apoptosis. Multivariable linear regression evaluated the interaction between urinary OH-PAH levels and apoptotic gene SNPs on apoptotic sperm, with genotype-stratified analyses. PAH exposure appeared to interact with SNPs in FasL rs763110, Fas rs2234767, and caspase-3 rs12108497 to jointly influence sperm cell apoptosis. Specifically, for the FasL rs763110, higher 9-OHFlu was associated with fewer viable sperm and more apoptotic sperm, and this association was more pronounced among CC genotype homozygotes. For the caspase-3 rs12108497, higher 2-OHFlu was associated with more dead sperm, and this association was significant among TC and TC/CC genotypes. These findings suggest that genetic variation in apoptosis-related genes may modify susceptibility to PAH-induced sperm apoptosis, highlighting the importance of gene–environment interactions in male reproductive toxicity.

## 1. Introduction

Globally, infertility affects approximately 50 million couples, with male factors accounting for approximately 30–50% of cases [1]. Male reproductive health is influenced by multiple factors, including age, lifestyle behaviors (such as smoking, excessive alcohol consumption, obesity, and sedentary behavior), psychological stress, clinical conditions (including varicocele, infections, and endocrine disorders), genetic susceptibility, and environmental exposures (pesticides, phosphates, plasticisers, etc.) [2]. Among these, exposure to environmental chemicals, particularly environmental endocrine disruptors (EDCs), is increasingly linked to impaired male reproductive outcomes [3]. Polycyclic aromatic hydrocarbons (PAHs), as a widely prevalent class of EDCs, are typical by-products of incomplete combustion of carbon-containing substances, commonly found in industrial emissions, barbecued and smoked foods, and occupational environments such as coking plants and asphalt paving, with dietary intake of high-temperature processed foods constituting nearly 90% of population exposure to PAHs [4,5]. Prolonged exposure to PAHs has been associated with a variety of adverse health outcomes, including carcinogenicity, mutagenicity, and reproductive toxicity. They enter the human body through multiple routes, including inhalation, ingestion, and skin contact [6], and have been detected in various biological matrices such as blood, urine, and breast milk [7,8]. Due to their persistence, lipophilicity, bioaccumulation, and endocrine-disrupting properties, PAHs hold significant toxicological importance. They may disrupt the normal functioning of reproductive, endocrine, and immune systems by interfering with steroid hormone synthesis and estrogen signaling pathways [9].

Increasing evidence suggests that PAHs may impair male reproductive function [10]. Animal studies have revealed that exposure to PAHs impairs male reproductive function in various amphibians, reptiles and semi-aquatic mammals [11,12]. Experimental data further indicate that PAHs and their metabolites can cross the blood-testis barrier and accumulate in the testes of rats, resulting in concomitant decreases in testosterone concentrations and impaired epididymal function [13]. In our previously conducted population-based studies, we observed that high levels of PAH metabolites in male urine may be associated with decreased sperm counts, concentrations, and percentage of normal morphology, as well as increased sperm DNA damage [14,15]. Mechanistically, metabolic activation of PAHs induces excessive generation of reactive oxygen species (ROS), leading to lipid peroxidation in sperm cell membranes [16], thereby reducing sperm motility and fertilization capacity. PAHs may also disrupt endocrine regulation of spermatogenesis through aryl hydrocarbon receptor–mediated interference with the hypothalamic–pituitary–testicular (HPT) axis [17]. Importantly, accumulating evidence indicates that PAHs can activate the death receptor pathway (such as the Fas/FasL system), culminating in the formation of the death-inducing signaling complex (DISC) and a consequent amplification of apoptotic signals [18]. Such dysregulated apoptosis is increasingly recognized as a key pathological mechanism underlying impaired spermatogenesis and reduced male fertility.

Under physiological conditions, apoptosis eliminates damaged or abnormal spermatozoa and maintains the equilibrium of sperm quality and quantity within the testes [19]. However, excessive or dysregulated apoptosis can disrupt this balance, resulting in impaired sperm production and reduced fertility [20]. This apoptotic imbalance is closely associated with alterations in gene regulation, hormonal homeostasis, and environmental physicochemical factors [21,22], and the extent of sperm apoptosis has therefore been widely recognized as a sensitive and informative biomarker for assessing male reproductive toxicity [23]. Among the apoptotic mechanisms involved, activation of the Fas/FasL-mediated extrinsic pathway has been identified as a critical mediator linking environmental insults to germ cell loss. Dysregulation of this pathway can trigger caspase-dependent cell death cascades, thereby amplifying spermatogenic damage. Fas is a member of the tumor necrosis factor receptor family and interacts with its natural ligand FasL to initiate an exogenous apoptotic pathway [24]. In the testis, Fas and FasL expression are primarily located in germ cells and Sertoli cells [18]. Furthermore, downstream activation of proteases such as caspase-3 by Fas can further propagate cell death signals [25]. Animal studies have demonstrated that caspase-3 activation is directly associated with sperm DNA damage and deteriorating semen parameters [26]. All of the above are considered key regulators of germ cell apoptosis activation. Research has revealed that single-nucleotide polymorphisms (SNPs) in the Fas, FasL, and caspase-3 genes can modulate their gene expression or function, and differential expression of these SNPs has been associated with human susceptibility to diseases such as hearing loss [27], recurrent miscarriage [28], and cancer [29,30] in humans. Collectively, these findings suggest that individual genetic variation may modulate vulnerability to environmentally induced reproductive toxicity, including that associated with PAH exposure.

Although increasing evidence links PAH exposure to impaired male reproductive function, the extent to which genetic susceptibility modifies these associations remains unclear. In particular, human data on gene-environment interactions between PAH exposure and polymorphisms in key apoptosis-related genes (such as Fas, FasL, and caspase-3) are notably lacking. To address this knowledge gap, this study aims to investigate whether functional polymorphisms in Fas (rs2234767), FasL (rs763110), and caspase-3 (rs12108497) modify the association between urinary PAH exposure and sperm apoptosis in men, thereby elucidating the role of genetic susceptibility in PAH-related reproductive toxicity.

## 2. Materials and Methods

### 2.1. Study Subjects

**Figure 1:**
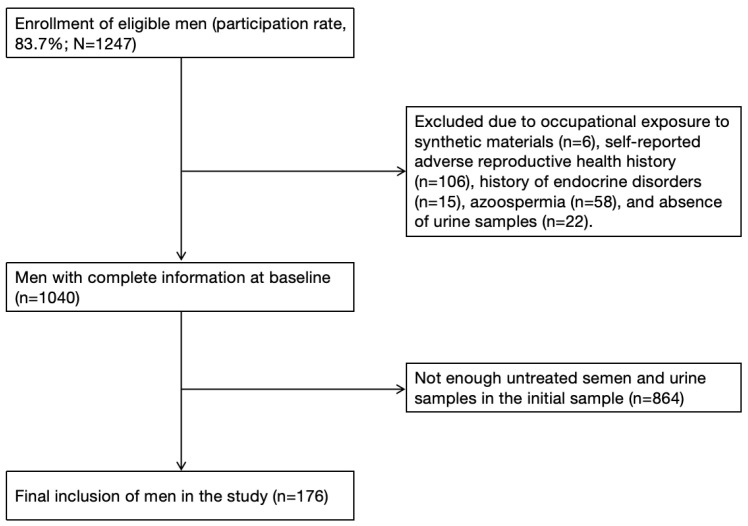
Flowchart for the study population.

This study is based on a cross-sectional survey of male reproductive function about environmental chemical exposure, conducted in Wuhan, China, from April to June 2013 [31]. The study protocol was approved by the Ethics Committee of Tongji Medical College, Huazhong University of Science and Technology ([2014] IEC S068). In brief, study participants were men in infertile couples seeking semen analysis, and all participants provided written informed consent form. During the study, each subject was required to provide two urine samples (average time interval: 4.4 ± 3.7 h) and complete a questionnaire containing demographic characteristics, history of occupational exposure, medical history, and lifestyle. Individuals with occupational exposure to synthetic materials (n = 6) and those reporting medical histories potentially affecting semen quality (n = 106), including epididymitis, vasectomy, varicocele, orchitis, epididymitis, testicular trauma, cryptorchidism; and endocrine disorders (n = 15), including diabetes mellitus or thyroid/adrenal disorders; 58 patients with azoospermia and 22 cases lacking urine samples were excluded. A total of 1040 eligible subjects were enrolled, 176 among them were selected for the present analyses because they had sufficient residual semen after routine semen analysis and chemical measurements (see Figure 1).

### 2.2. Semen Collection and Annexin V/PI Assay

Sperm apoptosis was measured by apoptosis kits coupled and flow cytometry as described in previous studies [31]. The results of sperm cell apoptosis parameters were expressed as percentages of total spermatozoa, including annexin V^−^/PI^−^ spermatozoa (viable cells), annexin V^+^/PI^−^ spermatozoa (apoptotic cells), and PI^+^ spermatozoa (dead cells). A 20 μL fresh semen sample was washed twice with 1 mL of phosphate-buffered saline (PBS, pH 7.2), and then the sample was resuspended in 500 μL of binding buffer to achieve a cell density of approximately 5 × 10^5^ cells/mL. 5 μL of Annexin V-FITC and 10 μL of propidium iodide (PI) were added to the buffer, and then the mixture was incubated in the dark at room temperature for 10 min.

### 2.3. Urinary OH-PAH Measures

To minimize the exposure classification arising from the short biological half-life (4.4–35 h) of OH-PAHs and intra-individual/inter-individual variations [32], each participant provided a first random urine sample between 8:30 and 11:30 on the survey day, followed by a second urine collection at least two hours later on the same day (mean interval: 4.4 ± 3.7 h). Urine samples were transported to the laboratory in ice packs and subsequently stored at −40 °C until analysis (storage duration < 6 months). Ten urinary OH-PAHs metabolites were quantified by gas chromatography-mass spectrometry (GC/MS): 1-hydroxynaphthalene (1-OHNa), 2-hydroxynaphthalene (2-OHNa), 2-hydroxyfluorene (2-OHFlu), 9-hydroxyfluorene (9-OHFlu), 1-hydroxyphenanthrene (1-OHPh), 2-hydroxyphenanthrene (2-OHPh), 3-hydroxyphenanthrene (3-OHPh), 4-hydroxyphenanthrene (4-OHPh), 9-hydroxyphenanthrene (9-OHPh), and 1-hydroxypyrene (1-OHP).

In brief, 1 mL urine sample was hydrolyzed at 37 °C for more than 12 h using β-glucuronidase/sulfatase (Sigma-Aldrich, Munich, Germany) (20 μL), followed by two extractions with n-hexane. The combined extract was evaporated to dryness, and the residue underwent derivatization with 100 μL of bistrimethylsilyl-trifluoroacetamide (BSTFA) at 45 °C for 90 min. The final extract was analyzed on an Agilent 6890 N gas chromatography coupled to 5975B mass spectrometry (Agilent Technologies, Santa Clara, CA, USA) [33]. Helium served as the carrier gas, with capillary column separation conducted at a constant flow rate of 1 mL/min. In splitless mode, the injection temperature was set to 300 °C. The optimized oven temperature program was as follows: initial temperature 60 °C (held for 3 min), ramped at 10 °C/min to 150 °C (held for 3 min), then ramped at 10 °C/min to 210 °C (held for 5 min), finally ramped at 10 °C/min to 320 °C (held for 2 min). The transfer line temperature is maintained at 280 °C. Mass spectrometry detection employs electron impact ionization mode with an ion source temperature of 300 °C. Data acquisition is conducted in the selected ion monitoring mode, scanning a mass-to-charge range of 50–500 [34].

Each analytical batch also included one blank sample, two quality control samples, and an unknown sample. Determination was performed using the ratio of internal standard peak area to sample concentration (R^2^), with all R^2^ values exceeding 0.999. The coefficient of variation for target analytes was below 10.0%, with mean spiked recoveries ranged from 77.7 to 116.0%. The limits of detection (LODs) for OH-PAH metabolites ranged from 0.03 to 0.18 μg/L. For concentrations below LOD, values were imputed as LOD/√2. Urinary creatinine was measured using Jaffe’s colorimetric method on an automated clinical chemistry analyzer. The concentrations of PAH metabolites were calibrated by urine creatinine and expressed as μg/g creatinine. The sum of 1-OHNa and 2-OHNa was referred to as ∑OHNa, the sum of 2-OHFlu and 9-OHFlu as ∑OHFlu, the sum of 1-OHPh, 2-OHPh, 3-OHPh, 4-OHPh, and 9-OHPh as ∑OHPh, and the sum of ∑OHNa, ∑OHFlu, ∑OHPh, and 1-OHP as ∑OH-PAHs.

### 2.4. Blood DNA Extraction and Genotyping

Extraction and sequencing of blood DNA were performed using the RelaxGene Blood DNA System (TIANGEN Biotech, Beijing, China) and real-time fluorescence quantitative polymerase chain reaction (TaqMan) SNP genotyping detection method (Applied Biosystems, Foster City, CA, USA), as detailed in our previous study [31]. We examined four SNPs associated with apoptosis-related genes, including the Fas rs1800682 and rs2234767, the FasL rs763110, and the caspase-3 rs12108497. Fluorescence signal detection and genotype calling were carried out on the ABI Prism 7900HT platform (Applied Biosystems, Foster City, CA, USA). Approximately 5% of samples were randomly selected for repeated genotyping with 99% concordance. All SNPs except the Fas rs1800682 conformed to Hardy–Weinberg equilibrium; consequently, the rs1800682 locus in the Fas gene was excluded from subsequent analyses.

### 2.5. Statistical Analysis

The participant demographics, urinary OH-PAH concentrations, and sperm cell apoptosis parameters were summarized as medians with interquartile ranges (P25, P75) for continuous variables due to their non-normal distribution, and as frequencies (n) with percentages (%) for categorical variables. The within-person variability of repeated urinary OH-PAH concentrations was assessed by calculating the intraclass correlation coefficient (ICC), which is the ratio of the between-person variance to the total variance.

To evaluate the associations between apoptosis-related SNPs (Fas, FasL, caspase-3), urinary OH-PAH concentrations and sperm apoptosis parameters, we constructed multivariable linear regression models. Both sperm apoptosis parameters and urinary OH-PAH concentrations underwent natural logarithmic transformation to improve normality. We assessed multiplicative interactions between urinary OH-PAHs and SNPs by including cross-product terms (OH-PAH × SNP) in the models and conducted genotype-stratified analyses. The percentage changes for the acquired estimated, and 95% confidence intervals were calculated and reported using the formula: (exp^|β|^ − 1) × 100. Genotype for each SNP was coded as value 0, 1, 2, corresponding to genotypes AA (wild-type homozygous), Aa (heterozygous), and aa (variant homozygous), respectively. Genotype coding was incorporated into the model as an ordered categorical variable. Based on the dominant inheritance model, genotypes Aa and aa were combined (combining genotypes) and compared with genotype AA [35].

The selection of confounding factors was guided by biological plausibility and statistical criteria [36,37,38]. A priori covariates included age (years), body mass index (BMI, kg/m^2^), duration of abstinence (≤2, 3, 4, 5, ≥6 days), and reproductive history (yes, no); Additional variables included smoking status (Never, Former, Current), drinking status (yes, no), income (monthly < 3000 yuan, 3000–6000 yuan, >6000 yuan), and education level (less than high school, high school and above), were retained if their inclusion led to a change in R^2^ of more than 10% [39]. A two-sided *p*-value < 0.05 was regarded as statistically significant. All data analyses were conducted in R 9.4 software.

## 3. Results

### 3.1. The Study Population’s Characteristics

Table 1 presents the demographic characteristics of the 176 participants in this study. The median age and body mass index (BMI) of the population were 31.0 years and 23.1 kg/m^2^, respectively. More than half of the participants had at least a high-school education, had a monthly income >3000 yuan, and were current smokers. Fewer than two-fifths of participants had a biological child as a father.

### 3.2. Distributions of Spermatozoa Apoptosis

Table 2 shows the sperm apoptosis parameters in this study population. The geometric means (GMs) for percentages of Annexin V^−^/PI^−^, Annexin V^+^/PI^−^, and PI^+^ spermatozoa were 73.5%, 12.25%, and 10.75%, respectively. Of these, the geometric means for early apoptosis and late apoptosis parameters in Annexin V^+^/PI^−^ spermatozoa were 2.7% and 8.95%.

### 3.3. Description of Polycyclic Aromatic Hydrocarbons (PAHs) Exposure

The 10 OH-PAH metabolites were detected in more than 90% of the urine on both occasions. Top three detections in the first urine were 2-OHNa, 9-OHFlu and 1-OHNa, and in the second urine were 2-OHNa, 9-OHFlu and 2-OHFlu. The ICC value for 1-OHNa concentration corrected for creatinine in duplicate urine samples was 0.84, was 0.45 for 3-OHPh, whereas most other OH-PAHs exhibited poor reproducibility (ICC < 0.40) (Table 3).

### 3.4. Associations Between Fas, FasL, and Caspase-3 Gene Polymorphisms and Urinary OH-PAHs Metabolites with Sperm Apoptosis

For FasL rs763110, compared to those with CC genotype, men with CT and CT/TT genotypes had higher propotion of Annexin PI^+^ spermatozoa by 27.63% (95% CI: 2.33%, 59.20%) and 28.79% (95% CI: 3.98%, 59.52%), respectively. No clear association was observed between apoptosis-related genes (Fas, FasL, or caspase-3) variants and the propotions of Annexin V^−^/PI^−^ or Annexin V^+^/PI^−^ apoptotic sperm in males (Figure S1, Table S2). The association between urinary OH-PAHs concentrations and percentage sperm apoptosis parameters in our small-sample study broadly aligned with our previous research [37], showing an inverse association between 9-OHPh and the proportion of Annexin V^−^/PI^−^ sperm (Table S1).

### 3.5. Gene-Environment Interactions: Apoptosis-Related SNPs × Urinary OH-PAHs

**Figure 2:**
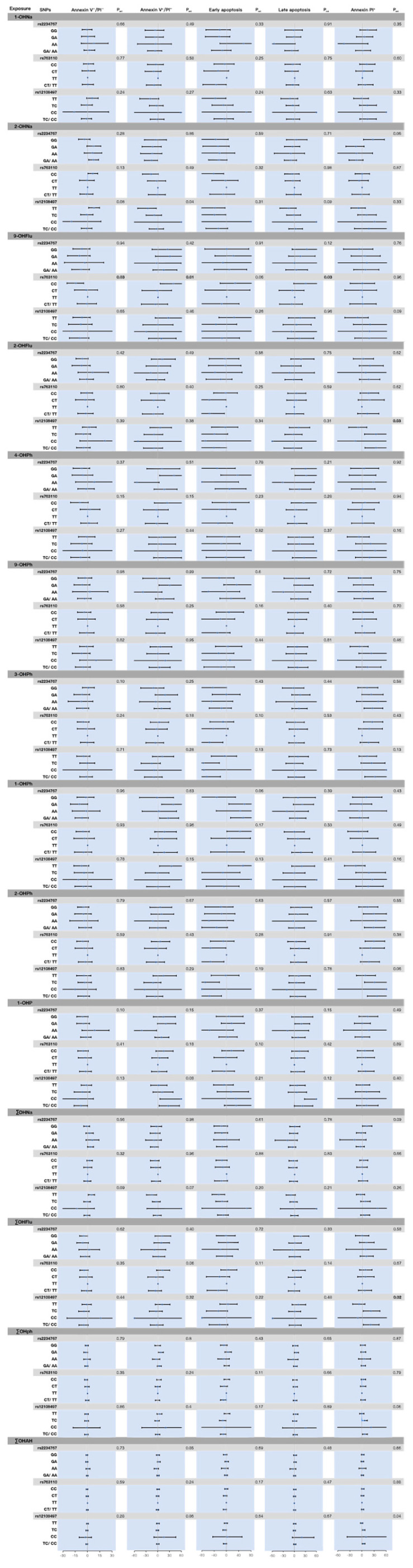
Percentage change in sperm apoptotic parameters associated with PAH exposure across genotypes. Multiple linear regression analyses were used, correcting for age, BMI, pregnacy, income, education, duration of abstinence, smoking status and alcohol consumption. The FasL rs763110 TT variant had insufficient sample numbers and was therefore excluded from analysis. Fas rs2234767 GG, FasL rs763110 CC, and caspase-3 rs12108497 TT are wild-type homozygote; Fas rs2234767 GA, FasL rs763110 CT, and caspase-3 rs12108497 TC are heterozygote; Fas rs2234767 AA, FasL rs763110 TT, and caspase-3 rs12108497 CC are Variant homozygote; Fas rs2234767 GA/AA, FasL rs763110 CT/TT, and caspase-3 rs12108497 TC/CC are combined genotype.

We observed a significant interaction between the FasL rs763110 SNP and urinary 9-OHFlu concentration in relation to sperm apoptosis: higher 9-OHFlu was associated with a lower proportion of viable cells (Annexin V^−^/PI^−^; p_int_ = 0.03) and a higher proportion of early apoptotic cells (Annexin V^+^/PI^−^; p_int_ = 0.01). In genotype-stratified analyses, these associations were confined to CC homozygotes with viable cell proportion (Annexin V^−^/PI^−^ spermatozoa) (−14.45%, 95% CI: −25.11%, −4.81%) and apoptotic cell proportion (Annexin V^+^/PI^−^ spermatozoa) (39.38%, 95% CI: 5.76%, 83.86%) (Figure 2, Table S3).

Furthermore, a significant interaction was observed between the caspase-3 rs12108497 and urinary 2-OHFlu concentration, where higher 2-OHFlu was associated with a greater proportion of dead sperm (PI^+^ spermatozoa) (p_int_ = 0.03). Among TC carriers and TC/CC carriers, urinary 2-OHFlu concentration was positively associated with the proportion of dead cells (PI^+^ spermatozoa) (34.72%, 95% CI: 6.50%, 70.23%; 34.99%, 95% CI: 7.47%, 69.55%, respectively). Consistent interaction patterns were observed for urinary ∑OHFlu (p_int_ = 0.02; 21.17%, 95% CI: 2.22%, 43.62%; 20.56%, 95% CI: 2.63%, 41.62%, respectively) and ∑OHAH (p_int_ = 0.04; 3.67%, 95% CI: 0.10%, 7.36%; 3.98%, 95% CI: 0.50%, 7.57%, respectively) (Figure 2, Table S3).

Although we observed an interaction between caspase-3 rs12108497 and the effect of urinary 2-OHNa on apoptotic cells (Annexin V^+^/PI^−^ spermatozoa), and an interaction between FasL rs763110 and urinary 9-OHFlu on the proportion of late-stage apoptotic cells, the corresponding genotype-specific associations were not statistically significant. At the Fas rs2234767 locus, the overall interaction term between OH-PAH and genotype did not reach statistical significance. However, among Fas rs2234767 GA carriers, urinary levels of 4-OHPh (40.92%, 95% CI: 4.29%, 90.41%), 9-OHPh (30.21%, 95% CI: 2.94%, 64.71%), and 1-OHPh (39.38%, 95% CI: 8.98%, 78.07%) were positively association with the percentage of sperm apoptosis (Annexin V^+^/PI^−^ spermatozoa) (Figure 2, Table S3).

## 4. Discussion

This study provides further evidence that genetic susceptibility may modulate the adverse effects of PAH exposure on male reproductive health, and we observed that polymorphisms in apoptosis-related genes, particularly Fas rs2234767, FasL rs763110, and caspase-3 rs12108497, may modify the association between urinary PAH metabolites and sperm apoptosis in a population of infertile men. Specifically, the FasL rs763110 interacted with urinary 9-OHFlu, corresponding to fewer viable cells and more early apoptotic cells at higher 9-OHFlu levels among CC homozygotes, whereas caspase-3 rs12108497 SNP interacted with urinary 2-OHFlu in relation to higher proportions of PI^+^ (dead/late apoptotic) cells. These gene–environment findings align with the a priori hypothesis that PAH-associated reproductive toxicity may be amplified in genetically susceptible subgroups.

Consistent with prior epidemiological studies, we observed directionally similar associations between urinary OH-PAHs and apoptosis parameters in this subset, although power was limited [14,40]. In our randomised small sample, it was once again suggest that genetic variation in key regulators of apoptotic signaling may influence individual susceptibility to PAH-induced reproductive toxicity. FasL and Fas binding are upstream of the apoptotic cascade response [25]. There is evidence that the promoter region of the FasL gene is highly polymorphic, and that the C and T alleles have different affinities for transcription factors [41]. The T allele may reduce basal expression of FasL compared to the C allele, affecting the biological activity of the FasL promoter [42] and thus sperm development [43], which is compatible with our observation that men carrying CT or CT/TT genotypes at FasL rs763110 had higher PI^+^ fractions than CC homozygotes. In addition, previous studies have shown that the Fas rs2234767 G allele also significantly reduces the binding of the transcription factor STAT1, which reduces Fas expression [44,45]. Caspase-3 is a key execution molecule in sperm apoptosis and its activity is also associated with genetic polymorphisms [46]. However, in subgroup analyses we also did not detect robust association of Fas or caspase-3 genotypes with sperm apoptosis, which may be due to limited sample size, phenotype variability, or context-dependent effects that are unmasked only under relevant exposures.

This study preliminarily found an interaction between the FasL rs763110 SNP and urinary 9-OHFlu concentration, which negatively influences live sperm counts and positively influences apoptotic sperm counts. The observed FasL rs763110 × 9-OHFlu interaction (strongest among CC homozygotes) is biologically plausible. PAHs can activate the aryl hydrocarbon receptor (AhR), perturbing endocrine signaling and inducing oxidative/genotoxic stress that converges on apoptosis pathways [47]. Animal studies also support this hypothesis: exposure to benzo[a]pyrene (B[a]P) significantly upregulates the expression of key apoptotic genes such as Fas and FasL in the testicular tissue of male rats [48]. These molecular changes are accompanied by phenotypic manifestations of reproductive toxicity, including reduced sperm count and decreased serum testosterone levels [48]. The FasL rs763110 C allele has been linked to higher promoter activity and protein expression [42]. Therefore, individuals homozygous for the C allele (CC genotype) may exhibit a more pronounced apoptotic response upon exposure to PAHs due to inherently enhanced FasL signaling, yielding larger decrements in viable cells and increases in apoptotic cells.

Previous toxicological evidence has demonstrated that PAH exposure can induce elevated high expression of caspase-3, thereby promoting germ cell apoptosis and contributing to sperm DNA damage [49]. Extending this evidence to a human population, we observed that the caspase-3 rs12108497 SNP × urinary 2-OHFlu concentration was associated with higher PI^+^ fractions among TC and TC/CC genotypes, with similar patterns for ∑OHFlu. Our findings further indicate that carrying the caspase-3 rs12108497 C allele may confer increased susceptibility to PAH-related sperm cell death, potentially via enhanced CASP3 activation or altered post-translational regulation [50]. Previous studies have suggested that carrying the C allele at the rs12108497 locus of the caspase-3 gene constitutes a risk factor for increased susceptibility to cancers such as gastric, colorectal, and hepatocellular carcinoma [51], supporting its functional relevance in regulating apoptotic processes.

In addition, accumulating evidence indicates that polymorphisms in other apoptosis-related genes may modify the reproductive effects of environmental toxicants. For example, a meta-analysis indicates that the SNP in the CYP1A1 gene of the cytochrome P450 enzyme superfamily interacts with PAH exposure to exert a positive influence on male infertility [10]. A study conducted in Jiangsu, China, targeting infertile males revealed a significant association between the XPA-4 G/A polymorphism and sperm DNA damage. Carriers of the XPA-4 AA homozygote exhibited markedly higher levels of sperm DNA damage compared to GG carriers. Furthermore, the XPA-4 G/A polymorphism was found to interact significantly with PAH exposure in influencing the risk of sperm DNA damage [52]. Consistent with these findings, our results suggest that genetic variation in apoptosis-related pathways may modulate individual vulnerability to PAH-induced reproductive toxicity. Notably, by focusing on Fas, FasL, and caspase-3, our study extends previous research by highlighting the role of apoptosis signaling in mediating gene–environment interactions affecting male reproductive health.

Despite these strengths, including the investigation of gene–environment interactions in relation to male reproductive toxicity, several limitations should be acknowledged. First, we used a dominant inheritance model in our study, which focused only on genetic polymorphisms, ignoring the differences between genotypically heterozygous and pure heterozygotes in the study. Second, although two urine samples were collected to reduce exposure misclassification, short-term variability in PAH exposure may still have introduced measurement error, potentially attenuating observed associations. Third, residual confounding (e.g., unmeasured dietary PAH sources, co-exposures, or clinical factors) was not comprehensively assessed. Fourth, information regarding individual’s underlying physical condition (such as potential chronic kidney disease, endocrine system diseases, etc.) mainly relies on self-reporting, which may introduce recall bias or result in misclassification. Fifth, the study population was recruited from a single infertility clinic and lacked a control group, which may limit the generalizability of the findings to the broader male population. Finally, the cross-sectional nature of this study cannot establish causality between PAH exposure, genetic polymorphisms, and sperm apoptosis. Future research needs to verify our findings by designing population-controlled or larger prospective cohorts and multi-center designs to further clarify the potential causal pathways.

## 5. Conclusions

In conclusion, our findings suggest that urinary PAH metabolites in combination with polymorphisms in Fas (rs2234767), FasL (rs763110), and CASP3 (rs12108497) are jointly associated with sperm apoptosis, with stronger adverse associations among genetically susceptible subgroups. These results underscore the importance of considering gene–environment interactions in evaluating male reproductive risk and highlight apoptosis as a critical biological pathway linking environmental exposures to impaired spermatogenesis. Future studies incorporating larger populations, longitudinal designs, and broader genomic coverage are warranted to confirm these findings and to further elucidate the mechanisms underlying environmentally induced male reproductive dysfunction.

## Figures and Tables

**Table 1:** Demographic characteristics of the study population.

Characteristics ^a^	Total Population (*n* = 176) ^b^
Age, year	31.0 (28.0, 36.0)
Body mass index, kg/m^2^	23.1 (21.1, 24.8)
Education	
Less than high school	73 (41.5)
High school and above	103 (58.5)
Income, yuan/month	
<3000	67 (38.0)
3000–6000	76 (43.2)
>6000	33 (18.8)
Alcohol consumption	
Yes	58 (33.0)
No	118 (67.0)
Smoking status	
Never	72 (40.9)
Former	16 (9.1)
Current	88 (50.0)
Abstinence duration, day	
≤2	20 (11.4)
3	52 (29.5)
4	37 (21.0)
5	25 (14.2)
≥6	42 (23.9)
Fathered a biological child	
Yes	69 (39.2)
No	107 (60.8)

^a^ 1 missing age. ^b^ For non-normally distributed continuous variables, data were expressed as median (P25, P75) using the Mann–Whitney U test; for categorical variables, data were expressed as n (%) using the chi-square test.

**Table 2:** Spermatozoa apoptosis parameters of the study population.

Spermatozoa Apoptosis	Mean	GM ^a^	Selected Percentile			
			5th	25th	75th	95th
Annexin V^−^/PI^−^ spermatozoa (%)	70.17	73.50	42.9	60.38	81.48	89.92
Annexin V^+^/PI^−^ spermatozoa (%)	16.72	12.25	3.80	7.80	22.20	42.96
Early apoptosis	5.36	2.70	0.40	1.20	5.43	20.88
Late apoptosis	11.36	8.95	2.69	5.80	13.88	32.27
PI^+^ spermatozoa (%)	13.10	10.75	2.79	7.00	17.73	32.48

^a^ GM is the geometric mean.

**Table 3:** Distribution of creatinine-corrected urinary OH-PAH concentrations (μg/g creatinine) in the study population ^a^.

OH-PAH ^b^	The First Urine Sample		The Second Urine Sample		ICCs ^d^
	% >LOD ^c^	Median (P25, P75)	% >LOD ^c^	Median (P25, P75)	
1-OHNa	99.43	2.29 (1.31, 4.03)	93.75	1.99 (0.84, 4.04)	0.84
2-OHNa	97.16	5.62 (3.19, 11.89)	94.89	5.42 (2.64, 10.97)	0.18
9-OHFlu	100.00	2.52 (1.86, 3.91)	99.43	2.66 (1.65, 4.29)	0.19
2-OHFlu	95.45	1.96 (1.21, 3.29)	94.32	2.63 (1.50, 3.92)	0.14
4-OHPh	100.00	1.10 (0.79, 1.60)	100.00	1.30 (0.82, 2.22)	0.12
9-OHPh	96.02	1.52 (0.96, 2.59)	100.00	1.75 (1.13, 3.02)	0.15
3-OHPh	98.86	0.71 (0.44, 1.17)	91.48	0.89 (0.42, 1.48)	0.45
1-OHPh	98.86	0.81 (0.55, 1.22)	94.32	0.62 (0.38, 1.13)	0.34
2-OHPh	93.75	0.83 (0.41, 1.53)	97.73	1.18 (0.62, 1.98)	0.35
1-OHP	92.61	0.65 (0.35, 1.27)	96.59	1.33 (0.67, 2.22)	0.05
∑OHNa	-	8.28 (5.16, 16.71)	-	7.83 (3.81, 14.90)	0.31
∑OHFlu	-	4.72 (3.37, 7.56)	-	5.26 (3.82, 7.90)	0.20
∑OHPh	-	5.26 (3.66, 7.86)	-	6.20 (4.16, 9.75)	0.30
∑OHAH	-	20.93 (14.69, 32.10)	-	22.70 (15.88, 36.13)	0.48

^a^ 1-OHNa, 1-hydroxynaphthalene; 2-OHNa, 2-hydroxynaphthalene; 2-OHFlu, 2-hydroxyfluorene; 9-OHFlu, 9-hydroxyfluorene; 1-OHPh, 1-hydroxyphenanthrene; 2-OHPh, 2-hydroxyphenanthrene; 3-OHPh, 3-hydroxyphenanthrene; 4-OHPh, 4-hydroxyphenanthrene; 9-OHPh, 9-hydroxyphenanthrene; and 1-OHP, 1-hydroxypyrene. The sum of 1-OHNa and 2-OHNa was referred to as ∑OHNa, the sum of 2-OHFlu and 9-OHFlu as ∑OHFlu, the sum of 1-OHPh, 2-OHPh, 3-OHPh, 4-OHPh, and 9-OHPh as ∑OHPh, and the sum of ∑OHNa, ∑OHFlu, ∑OHPh, and 1-OHP as ∑OH-PAHs. ^b^ Creatinine-adjusted urinary TCAA concentrations (μg/g) were calculated by dividing the crude target compound concentrations (μg/L) by creatinine concentrations (g/L) to correct for urine dilution. ^c^ LOD, limit of detection, “-” denotes no LOD. ^d^ ICCs, intraclass correlation coefficients.

## Data Availability

The data presented in this study are available on request from the corresponding author.

## Supplementary Materials

The following supporting information can be downloaded at: https://www.mdpi.com/article/10.3390/toxics14010050/s1, Table S1: Percentage change in spermatozoa apoptosi and PAH exposure; Table S2: Percentage change for spermatozoa apoptosis with Fas, FasL and caspase 3 genotypes; Table S3: Percentage change in sperm apoptotic parameters associated with PAH exposure across genotypes; Figure S1: Percentage change for spermatozoa apoptosis with Fas, FasL and caspase-3 genotypes.

## Abbreviations

The following abbreviations are used in this manuscript:

PAH	polymorphisms and urinary polycyclic aromatic hydrocarbon
SNPs	single nucleotide polymorphisms
EDCs	environmental endocrine disruptors
ICC	intraclass correlation coefficient
BMI	body mass index
GMs	geometric means
