# Supplementary material for: Gene–Environment Interactions of Apoptosis-Related Polymorphisms and Urinary Polycyclic Aromatic Hydrocarbon (PAH) Metabolites in Relation to Sperm Cell Apoptosis Among Men Attending Infertility Clinics
Source: Toxics. 2025 Dec 31;14(1):50. doi: 10.3390/toxics14010050 (PMC12846204; doi:10.3390/toxics14010050)
Supplement: Supplementary file 1 [file toxics-14-00050-s001.zip › toxics-4021755-Supplementary.pdf]

Supplementary Information

Gene–environment interactions of apoptosis-related polymorphisms and urinary polycyclic aromatic hydrocarbon (PAH) metabolites in relation to sperm cell apoptosis among men attending infertility clinics

Shiting Yi [1, 2], Sitong Lin [1, 2], Jiabin Xie [3], Zhihong Yang [3], Junxia You [3], Ximei Zhong [3], Hui Yang [2], Haiqing Lin [2], Qian Wang [2], Yajie Gong [2], Pan Yang [4], Yan Bai [2 *], Yingjun Chena [1, 2 *]

Contents	Pages
Table S1 Percentage change in spermatozoa apoptosi and PAH exposure.	S1
Table S2 Percentage change for spermatozoa apoptosis with Fas, FasL and caspase 3 genotypes.	S2-S3
Table S3 Percentage change in sperm apoptotic parameters associated with PAH exposure across genotypes.	S4-S16
Figure S1 Percentage change for spermatozoa apoptosis with Fas, FasL and caspase-3 genotypes.	S17

Table S1 Percentage change in spermatozoa apoptosi and PAH exposure [a].

Metabolites	Annexin V^-/PI^- spermatozoa (%)<br>β, 95%CI	Annexin V^+/PI^- spermatozoa (%)<br>β, 95%CI	Early apoptosis<br>β, 95%CI	Late apoptosis<br>β, 95%CI	PI^+ spermatozoa (%)<br>β, 95%CI
1-OHNa	1.11(−3.25, 5.55)	−9.20(−23.49, 3.67)	−21.05(−46.81, 0.20)	−6.82(−20.92, 5.87)	7.68(−4.60, 21.17)
2-OHNa	3.05(−1.31, 7.57)	−13.20(−28.15, 0)	−29.69(−57.3, −6.93)	−5.87(−19.96, 6.93)	−0.30(−13.09, 12.52)
9-OHFlu	-8.22(-15.95, -1.01)	16.77(-4.81, 42.76)	4.39(-31.65, 43.48)	13.20(-8.00, 38.40)	1.71(-19.36, 23.61)
2-OHFlu	-1.92(-7.47, 3.46)	-1.61(-18.41, 14.68)	-19.36(-51.74, 6.40)	3.67(-12.41, 20.80)	13.20(-2.22, 30.87)
4-OHPh	-3.98(-11.18, 2.94)	10.63(-9.97, 34.45)	-9.31(-48.59, 24.36)	16.53(-4.19, 41.48)	4.19(-15.84, 25.73)
9-OHPh	-2.84(-8.76, 2.84)	3.87(-13.2, 22.26)	-6.08(-36.89, 21.77)	6.40(-10.52, 24.98)	2.33(-14.22, 19.60)
3-OHPh	-3.05(-8.33, 2.12)	0.30(-15.49, 16.07)	-28.02(-60.80, -2.02)	5.34(-9.86, 21.90)	21.05(5.44, 38.96)
1-OHPh	−5.97(−12.3, 0.10)	16.18(−2.02, 37.71)	23.24(−6.08, 61.12)	7.47(−10.41, 27.38)	−1.41(−19.48, 16.30)
2-OHPh	−4.50(−9.86, 0.70)	2.02(−13.54, 18.18)	−26.74(−59.36, −0.90)	8.65(−6.61, 25.73)	23.00(7.25, 41.20)
1-OHP	−3.98(−8.65, 0.6)	10.74(−2.84, 25.99)	5.65(−16.18, 29.69)	12.19(−1.41, 27.63)	6.08(−6.82, 20.20)
∑OHNa	1.41(−1.11, 3.98)	−7.47(−15.60, 0)	−16.65(−30.47, −4.29)	−4.29(−12.19, 3.05)	2.43(−4.71, 9.86)
∑OHFlu	−3.15(−7.04, 0.50)	3.56(−7.47, 15.26)	−7.68(−27.25, 9.75)	5.34(−5.55, 17.12)	6.72(−3.77, 18.18)
∑OHPh	−1.21(−2.53, 0.20)	1.61(−2.43, 5.76)	−3.25(−9.86, 3.15)	2.43(−1.61, 6.50)	3.25(−0.60, 7.25)
∑OHAH	−0.50(−1.21, 0.30)	0.30(−1.92, 2.53)	−2.63(−6.18, 0.90)	0.90(−1.31, 3.15)	1.71(−0.50, 3.87)

[a] Multiple linear regression analyses were used, correcting for age, BMI, pregnacy, income, education, duration of abstinence, smoking status and alcohol consumption.

Table S2 Percentage change for spermatozoa apoptosis with Fas, FasL and caspase 3 genotypes [a].

Gene	Genotypes	N(%)	Annexin V^-/PI^- spermatozoa (%)	Annexin V^+/PI^- spermatozoa (%)	Early apoptosis	Late apoptosis	PI^+ spermatozoa (%)
			β, 95%CI	β, 95%CI	β, 95%CI	β, 95%CI	β, 95%CI
Fas							
rs2234767	GG [b]	73 (41.5)	Reference	Reference	Reference	Reference	Reference
	GA [c]	75 (42.6)	−1.21(−10.41, 7.79)	4.39(−23.37, 34.58)	6.18(−40.21, 57.93)	6.82(−20.44, 37.44)	5.55(−20.68, 34.58)
	AA [d]	28 (15.9)	−1.61(−13.77, 10.08)	8.33(−28.02, 50.08)	15.60(−44.48, 92.90)	−4.81(−45.06, 32.05)	−5.97(−44.92, 29.05)
	GA/ AA [e]	103 (58.5)	−1.31(−9.86, 6.93)	5.55(−19.48, 33.24)	8.87(−32.31, 56.99)	3.25(−22.14, 30.21)	2.12(−22.51, 27.63)
FasL							
rs763110	CC [b]	103 (58.5)	Reference	Reference	Reference	Reference	Reference
	CT [c]	64 (36.4)	−2.84(−11.52, 5.34)	4.81(−20.56, 32.58)	3.36(−39.51, 49.03)	11.40(−13.31, 40.64)	27.63(2.33, 59.20)
	TT [d]	9 (5.1)	2.53(−16.53, 22.38)	−7.04(−79.32, 56.36)	−71.26(−283.44, 30.60)	20.44(−38.68, 100.97)	38.82(−17.23, 125.92)
	CT/ TT [e]	73 (41.5)	−2.33(−10.63, 5.76)	3.56(−21.17, 29.95)	−2.94(−47.11, 38.68)	12.41(−11.52, 40.78)	28.79(3.98, 59.52)
Caspase 3							
rs12108497	TT [b]	89 (50.6)	Reference	Reference	Reference	Reference	Reference
	TC [c]	70 (39.8)	0.50(−7.79, 8.87)	0(−26.24, 26.24)	8.22(−33.24, 56.05)	−4.19(−31.39, 21.05)	−1.92(−27.25, 22.38)
	CC [d]	17 (9.6)	10.41(−3.77, 26.62)	−20.44(−79.14, 23.49)	−8.76(−103.20, 71.77)	−14.68(−70.57, 29.56)	−44.05(−110.22, 1.31)
	TC/ CC [e]	87 (49.4)	2.22(−5.55, 10.30)	−3.56(−29.05, 20.44)	5.02(−34.58, 48.44)	−6.08(−32.05, 17.35)	−8.65(−34.18, 13.66)

[a] Multiple linear regression analyses were used, correcting for age, BMI, pregnacy, income, education, duration of abstinence, smoking status and alcohol consumption.
[b] Wild-type homozygote.

[c] Heterozygote.
[d] Variant homozygote.
[e] Combined genotype.

Table S3 Percentage change in sperm apoptotic parameters associated with PAH exposure across genotypes [a].

Metabolites	Gene	Genotypes	Annexin V^-/PI^- spermatozoa (%) β, 95%CI	Annexin V^+/PI^- spermatozoa [b] (%) β, 95%CI	Early apoptosis β, 95%CI	Late apoptosis β, 95%CI	PI^+ spermatozoa (%) β, 95%CI
1-OHNa							
	rs2234767						
		Interaction	0.66	0.49	0.33	0.91	0.35
		Stratified					
		GG	−0.70(−6.72, 5.34)	−7.36(−32.84, 15.26)	−22.51(−67.87, 11.96)	−2.33(−26.74, 21.17)	12.75(−6.18, 34.99)
		GA	0.40(−7.57, 8.55)	−7.47(−31.65, 13.88)	−23.86(−69.22, 10.41)	−2.22(−22.51, 17.23)	4.81(−14.80, 26.11)
		AA	7.68(−7.90, 25.11)	−6.40(−52.04, 34.31)	55.12(−22.02, 193.29)	−56.05(−146.95, 1.31)	−45.50(−141.33, 13.88)
		GA/ AA	2.02(−4.50, 8.76)	−7.36(−26.49, 9.86)	−14.22(−49.33, 14.34)	−7.47(−26.74, 9.86)	1.51(−17.00, 20.44)
	rs763110						
		Interaction	0.77	0.58	0.25	0.75	0.60
		Stratified					
		CC	1.01(−5.02, 7.25)	−5.34(−26.49, 14.00)	−6.29(−40.92, 24.61)	−4.71(−26.49, 15.37)	10.85(−6.82, 31.26)
		CT	1.01(−6.82, 8.98)	−11.07(−33.24, 8.11)	−27.89(−74.37, 6.50)	−7.14(−26.49, 10.30)	3.98(−16.42, 25.73)
		TT	/	/	/	/	/
		CT/ TT	0.30(−6.50, 7.14)	−9.64(−29.69, 7.90)	−33.91(−77.36, −1.01)	−5.23(−23.24, 11.29)	7.04(−10.30, 26.49)
	rs12108497						
		Interaction	0.24	0.27	0.24	0.63	0.33
		Stratified					
		TT	5.55(−1.21, 12.75)	−19.72(−46.23, 1.92)	−37.16(−83.49, −2.53)	−12.41(−37.30, 8.65)	−2.84(−21.77, 15.03)
		TC	−0.90(−7.79, 5.87)	−3.67(−21.77, 13.31)	−10.52(−47.70, 20.92)	−5.13(−23.24, 11.63)	12.19(−7.14, 34.85)

		CC	−16.42(−70.23, 25.61)	57.93(−159.09, 546.24)	64.05(−362.28, 1144.10)	71.77(−169.66, 695.66)	88.33(−352.22, 1502.26)
		TC/ CC	−1.31(−7.68, 4.81)	−1.82(−19.84, 15.72)	−7.57(−41.06, 21.90)	−3.56(−22.02, 13.88)	12.41(−6.50, 34.45)
2-OHNa							
	rs2234767						
		Interaction	0.28	0.86	0.59	0.71	0.06
		Stratified					
		GG	−3.77(−10.96, 3.05)	−5.02(−34.45, 21.90)	−35.53(−94.45, 5.97)	5.13(−21.90, 34.58)	26.74(3.56, 55.12)
		GA	7.36(−0.90, 16.18)	−13.54(−40.21, 8.76)	−27.38(−76.83, 8.98)	−5.23(−27.38, 14.91)	−15.60(−40.07, 4.81)
		AA	6.18(−4.08, 17.35)	−14.34(−44.34, 10.52)	−22.88(−91.55, 27.00)	−10.19(−54.96, 27.63)	−13.77(−62.91, 25.86)
		GA/ AA	7.14(1.31, 13.43)	−16.07(−34.45, −0.30)	−24.98(−58.72, 1.71)	−9.86(−27.63, 5.65)	−13.66(−32.45, 2.53)
	rs763110						
		Interaction	0.13	0.49	0.32	0.98	0.87
		Stratified					
		CC	6.18(0.50, 12.30)	−17.35(−39.51, 1.21)	−41.34(−83.49, −8.98)	−5.97(−27.00, 13.09)	0.40(−17.23, 18.29)
		CT	−0.70(−9.75, 8.22)	−2.74(−26.49, 19.84)	−3.36(−47.70, 38.13)	−4.39(−25.99, 15.60)	−7.25(−32.84, 15.49)
		TT	/	/	/	/	/
		CT/ TT	−1.21(−9.31, 6.72)	−2.02(−24.23, 19.36)	−10.41(−54.65, 26.87)	−1.82(−22.38, 18.18)	−3.25(−25.36, 17.70)
	rs12108497						
		Interaction	0.08	0.04	0.31	0.09	0.33
		Stratified					
		TT	7.79(1.31, 14.68)	−25.48(−51.44, −3.87)	−37.99(−82.03, −4.50)	−15.60(−39.79, 4.60)	−8.65(−27.51, 8.00)
		TC	−0.90(−8.55, 6.61)	−9.09(−30.08, 9.42)	−38.96(−89.46, −1.92)	−1.71(−21.41, 17.23)	10.41(−11.07, 35.39)
		CC	−19.72(−66.53, 16.18)	126.14(−31.52, 573.29)	134.90(−137.03, 1207.89)	133.03(−47.11, 699.65)	30.60(−486.50, 900.41)
		TC/ CC	−1.11(−7.68, 5.34)	−3.36(−22.38, 14.68)	−26.62(−67.03, 4.08)	3.05(−15.14, 22.26)	16.77(−18.89, 62.09)
9-OHFlu							
	rs2234767						

	Interaction	0.94	0.42	0.91	0.12	0.76
	Stratified					
	GG	−7.04(−17.94, 2.94)	24.11(−15.14, 77.18)	0.30(−70.92, 72.12)	33.24(−7.04, 89.84)	−8.00(−46.81, 25.86)
	GA	−9.97(−24.48, 3.05)	30.34(−6.61, 80.94)	21.90(−37.99, 105.24)	15.49(−16.53, 55.43)	6.08(−28.02, 44.05)
	AA	−3.46(−28.15, 19.60)	−3.25(−69.55, 58.88)	−12.08(−184.06, 126.14)	−0.60(−103.40, 100.97)	10.85(−89.84, 133.26)
	GA/ AA	−8.11(−19.36, 2.22)	14.45(−13.09, 48.14)	8.44(−40.64, 65.53)	4.29(−24.48, 35.39)	7.57(−21.65, 40.78)
rs763110						
	Interaction	0.03	0.01	0.06	0.03	0.96
	Stratified					
	CC	−14.45(−25.11, −4.81)	39.38(5.76, 83.86)	27.63(−21.17, 97.19)	29.95(−2.74, 73.50)	0(−30.34, 30.34)
	CT	−1.71(−16.07, 12.19)	−2.33(−41.06, 34.72)	1.51(−70.74, 75.77)	−6.61(−42.48, 25.36)	−3.05(−43.48, 35.26)
	TT	/	/	/	/	/
	CT/ TT	−0.90(−12.75, 10.85)	−6.82(−42.19, 24.61)	−16.42(−90.03, 40.21)	−7.79(−40.92, 21.17)	1.21(−31.13, 34.31)
rs12108497						
	Interaction	0.65	0.46	0.26	0.96	0.09
	Stratified					
	TT	−6.82(−17.94, 3.25)	26.11(−6.82, 69.89)	24.23(−25.11, 93.09)	15.37(−16.65, 55.27)	−11.85(−43.33, 14.57)
	TC	−8.22(−23.37, 5.44)	−0.10(−38.54, 38.40)	−34.85(−141.57, 32.84)	4.92(−31.52, 44.92)	18.89(−22.14, 72.81)
	CC	−19.48(−88.89, 32.18)	−29.69(−652.33, 346.83)	−127.28(−2314.31, 367.39)	−9.09(−665.23, 543.02)	17.70(−1131.72, 1604.74)
	TC/ CC	−9.64(−22.14, 1.61)	8.55(−23.99, 46.08)	−15.26(−88.51, 41.91)	12.75(−19.48, 52.04)	16.77(−18.89, 62.09)
2-OHFlu						
rs2234767						
	Interaction	0.42	0.49	0.58	0.75	0.62
	Stratified					
	GG	−6.40(−14.11, 0.80)	11.40(−16.53, 44.77)	−4.92(−55.43, 41.06)	18.53(−9.42, 53.73)	3.25(−21.17, 29.05)
	GA	−7.36(−18.89, 3.15)	1.21(−29.69, 32.98)	−3.46(−58.25, 47.70)	−7.14(−36.48, 18.89)	19.72(−6.72, 52.96)

	AA	6.08(−11.52, 25.61)	−17.82(−73.67, 25.11)	−37.16(−184.34, 51.13)	−13.09(−97.98, 54.96)	9.53(−65.53, 98.58)
	GA/ AA	−1.11(−9.64, 7.25)	−4.81(−29.05, 17.47)	−18.41(−65.86, 18.29)	−1.71(−25.48, 21.17)	16.65(−6.18, 44.48)
rs763110						
	Interaction	0.80	0.40	0.25	0.59	0.62
	Stratified					
	CC	−0.90(−8.11, 6.18)	4.08(−18.89, 28.79)	−1.92(−41.48, 36.21)	5.65(−17.94, 31.52)	16.30(−4.60, 41.48)
	CT	−2.94(−14.68, 8.11)	−9.97(−42.48, 17.82)	−55.43(−138.93, −1.21)	5.02(−20.56, 32.84)	19.24(−9.31, 55.43)
	TT	/	/	/	/	/
	CT/ TT	−2.22(−11.85, 7.04)	−10.85(−39.38, 13.54)	−55.43(−127.50, −6.29)	1.41(−22.38, 25.86)	13.20(−10.74, 41.91)
rs12108497						
	Interaction	0.39	0.38	0.34	0.31	0.03
	Stratified					
	TT	2.02(−6.18, 10.52)	5.97(−20.32, 35.12)	−3.87(−48.44, 37.58)	11.96(−13.43, 42.19)	−8.87(−32.98, 12.19)
	TC	−5.02(−14.57, 3.77)	−9.42(−35.26, 12.98)	−39.38(−102.99, 4.50)	−1.82(−25.73, 21.41)	34.72(6.50, 70.23)
	CC	21.05(−9.31, 60.16)	−50.38(−378.27, 111.28)	−67.87(−771.46, 209.57)	−61.12(−467.46, 118.58)	−16.53(−635.96, 441.95)
	TC/ CC	−4.71(−13.31, 3.25)	−10.19(−36.48, 12.41)	−40.78(−99.37, 0.50)	−3.36(−28.27, 20.20)	34.99(7.47, 69.55)
4-OHPh						
rs2234767						
	Interaction	0.37	0.51	0.78	0.21	0.92
	Stratified					
	GG	−7.14(−17.59, 2.53)	10.30(−28.40, 56.05)	−30.73(−119.24, 28.27)	30.21(−8.22, 83.49)	4.60(−28.66, 40.78)
	GA	−5.65(−18.89, 6.40)	40.92(4.29, 90.41)	36.21(−18.89, 120.34)	32.18(0.90, 73.33)	0.80(−32.05, 34.04)
	AA	8.11(−14.68, 34.04)	−53.57(−143.27, 3.15)	−91.17(−368.8, 28.27)	−31.92(−167.51, 53.57)	28.02(−65.70, 171.83)
	GA/ AA	−1.71(−11.96, 8.22)	16.30(−9.97, 48.88)	9.31(−37.03, 63.72)	12.86(−13.54, 44.77)	1.92(−27.00, 31.78)
rs763110						
	Interaction	0.15	0.15	0.23	0.28	0.94

	Stratified					
	CC	−9.42(−20.68, 0.70)	23.74(−9.20, 67.20)	8.76(−46.81, 73.67)	25.11(−8.98, 70.74)	6.82(−24.11, 41.76)
	CT	2.74(−8.98, 15.03)	−3.87(−36.75, 26.62)	−22.02(−94.64, 30.73)	4.39(−22.75, 33.78)	−2.22(−35.66, 29.95)
	TT	/	/	/	/	/
	CT/ TT	1.71(−8.33, 12.08)	−2.94(−32.05, 24.73)	−27.38(−94.64, 19.84)	6.08(−19.01, 33.91)	1.31(−26.24, 29.56)
rs12108497						
	Interaction	0.27	0.44	0.82	0.37	0.16
	Stratified					
	TT	−0.90(−11.07, 9.09)	8.44(−23.24, 45.06)	−7.04(−64.05, 43.19)	13.43(−17.35, 51.13)	−8.00(−37.16, 17.59)
	TC	−6.93(−20.44, 5.44)	8.33(−23.74, 45.35)	−17.47(−99.77, 44.63)	13.43(−17.82, 51.74)	20.44(−16.30, 68.71)
	CC	−6.08(−88.70, 67.87)	208.64(−90.22, 1711.97)	89.84(−841.21, 3295.38)	291.57(−63.39, 2402.81)	31.78(−1672.54, 2978.42)
	TC/ CC	−8.44(−20.56, 2.53)	20.08(−11.07, 60.00)	−2.63(−66.20, 57.78)	25.86(−5.97, 67.87)	18.06(−16.65, 62.58)
9-OHPh						
rs2234767						
	Interaction	0.98	0.99	0.60	0.72	0.75
	Stratified					
	GG	−3.05(−11.63, 5.13)	−3.25(−38.54, 30.08)	−23.86(−91.75, 24.98)	4.60(−28.40, 40.49)	−4.50(−34.31, 22.88)
	GA	−6.40(−16.53, 2.94)	30.21(2.94, 64.71)	33.38(−8.87, 93.67)	21.90(−1.41, 50.68)	2.53(−21.77, 28.15)
	AA	2.74(−18.29, 24.86)	−38.26(−111.28, 10.52)	−58.72(−260.74, 43.33)	−30.21(−144.73, 44.34)	15.84(−70.23, 128.42)
	GA/ AA	−3.67(−12.19, 4.39)	15.26(−6.29, 41.20)	14.80(−21.41, 60.16)	11.74(−9.86, 37.16)	3.25(−19.84, 27.63)
rs763110						
	Interaction	0.58	0.25	0.16	0.40	0.70
	Stratified					
	CC	−3.87(−12.19, 4.08)	8.00(−17.47, 37.16)	7.90(−33.78, 55.89)	8.22(−18.18, 38.4)	2.22(−22.14, 27.63)
	CT	−1.11(−11.96, 9.42)	−0.90(−29.18, 26.74)	−19.48(−81.67, 27.12)	6.93(−16.77, 33.51)	5.13(−22.63, 35.53)
	TT	/	/	/	/	/

	CT/ TT	−0.80(−9.75, 8.00)	−5.02(−30.60, 18.41)	−29.30(−87.01, 11.85)	1.41(−20.92, 24.48)	6.08(−16.88, 31.52)
rs12108497						
	Interaction	0.82	0.95	0.44	0.81	0.46
	Stratified					
	TT	−1.21(−9.64, 7.04)	6.40(−19.72, 35.66)	5.13(−35.93, 50.23)	7.04(−18.77, 36.07)	−6.29(−29.82, 14.91)
	TC	−7.25(−18.53, 3.05)	−0.10(−28.27, 28.02)	−25.61(−95.81, 24.11)	4.08(−22.75, 33.11)	22.75(−8.00, 62.74)
	CC	10.41(−9.64, 33.51)	19.12(−78.96, 153.96)	11.52(−166.18, 231.35)	18.53(−94.64, 173.46)	−76.12(−391.36, 58.57)
	TC/ CC	−5.34(−14.80, 3.46)	3.67(−22.14, 31.26)	−17.47(−73.33, 25.73)	8.11(−17.23, 37.03)	13.2(−14.57, 46.81)
3-OHPh						
rs2234767						
	Interaction	0.10	0.25	0.43	0.44	0.58
	Stratified					
	GG	1.01(−6.18, 8.33)	−11.40(−43.62, 15.72)	−44.2(−108.76, 0.50)	−2.63(−32.71, 25.86)	12.19(−10.63, 39.24)
	GA	−6.61(−16.65, 2.53)	18.53(−6.82, 50.23)	−0.50(−46.23, 44.63)	15.37(−7.14, 42.48)	22.75(−0.80, 52.04)
	AA	−7.79(−23.61, 6.50)	−5.55(−46.67, 31.65)	−49.03(−168.32, 20.8)	6.40(−50.08, 69.89)	40.07(−14.00, 123.67)
	GA/ AA	−5.76(−13.43, 1.41)	9.42(−9.64, 31.39)	−15.84(−55.89, 16.07)	12.3(−6.93, 34.72)	25.86(4.71, 51.29)
rs763110						
	Interaction	0.24	0.18	0.10	0.53	0.43
	Stratified					
	CC	−5.23(−12.52, 1.71)	3.36(−19.24, 27.38)	−11.40(−53.57, 23.86)	2.33(−21.29, 27.00)	25.23(3.67, 51.29)
	CT	−1.82(−12.52, 8.55)	−2.74(−30.87, 23.99)	−42.33(−112.97, 5.23)	8.98(−14.11, 35.53)	17.59(−8.76, 50.38)
	TT	/	/	/	/	/
	CT/ TT	−0.40(−8.65, 7.79)	−6.50(−30.34, 15.03)	−55.27(−115.98, −11.63)	4.92(−15.26, 26.74)	15.95(−4.92, 41.20)
rs12108497						
	Interaction	0.71	0.28	0.13	0.73	0.13
	Stratified					

	TT	−4.92(−13.20, 2.94)	11.18(−13.66, 40.49)	−5.87(−49.48, 33.38)	9.75(−14.91, 38.26)	12.30(−7.90, 36.07)
	TC	−0.30(−9.42, 8.76)	−15.72(−42.62, 6.40)	−75.24(−149.68, −22.88)	0.20(−23.12, 23.61)	23.37(−2.94, 56.67)
	CC	1.41(−34.31, 37.99)	−8.87(−232.01, 180.11)	−73.15(−657.61, 152.69)	3.25(−230.36, 252.19)	78.60(−172.64, 770.59)
	TC/ CC	−1.61(−9.42, 5.97)	−9.86(−34.18, 11.18)	−63.72(−124.12, −19.60)	4.19(−17.47, 27.63)	32.05(6.61, 63.56)
1-OHPh						
rs2234767						
	Interaction	0.96	0.63	0.06	0.39	0.43
	Stratified					
	GG	−2.33(−12.86, 7.79)	−2.63(−47.11, 39.65)	−32.18(−125.47, 28.92)	9.31(−31.26, 56.67)	9.42(−24.11, 48.59)
	GA	−9.97(−20.92, 0.10)	39.38(8.98, 78.07)	57.93(7.14, 132.80)	20.92(−3.67, 51.74)	−2.63(−29.95, 23.49)
	AA	−1.51(−18.77, 15.14)	2.43(−40.35, 47.11)	44.34(−33.64, 178.71)	−20.80(−100.37, 37.44)	−7.25(−84.78, 60.80)
	GA/ AA	−6.93(−15.37, 0.90)	25.99(3.87, 52.81)	53.73(12.64, 110.01)	7.25(−13.88, 30.87)	−6.72(−31.13, 15.14)
rs763110						
	Interaction	0.93	0.96	0.17	0.33	0.49
	Stratified					
	CC	−5.65(−14.00, 2.02)	16.53(−8.33, 46.96)	43.48(0.80, 104.21)	−0.70(−28.27, 26.49)	−9.42(−36.07, 13.54)
	CT	−3.87(−16.88, 8.33)	11.07(−19.84, 47.99)	8.65(−50.53, 77.71)	8.65(−19.24, 40.92)	3.25(−30.34, 38.96)
	TT	/	/	/	/	/
	CT/ TT	−5.13(−15.95, 5.02)	15.95(−10.96, 49.18)	1.71(−52.04, 57.46)	17.00(−8.11, 47.99)	3.67(−23.99, 33.24)
rs12108497						
	Interaction	0.78	0.15	0.13	0.41	0.16
	Stratified					
	TT	−7.25(−16.88, 1.51)	34.31(4.08, 73.33)	53.11(5.23, 122.78)	19.60(−8.11, 54.65)	−16.18(−44.05, 6.72)
	TC	−4.39(−16.30, 6.72)	−1.71(−32.31, 28.02)	−12.19(−80.58, 43.48)	−1.31(−31.65, 28.15)	16.30(−16.30, 57.46)
	CC	−11.29(−62.09, 30.87)	121.44(−45.94, 614.92)	159.61(−121.67, 1395.43)	144.00(−46.37, 771.46)	24.61(−570.60, 941.24)
	TC/ CC	−5.13(−15.49, 4.50)	0.10(−29.05, 29.43)	−8.76(−66.53, 40.64)	0.20(−29.18, 29.82)	14.34(−16.07, 51.74)

2-OHPh							
	rs2234767						
		Interaction	0.79	0.67	0.63	0.57	0.55
		Stratified					
		GG	−5.13(−12.19, 1.61)	5.97(−20.44, 35.26)	−18.41(−70.23, 21.41)	15.37(−10.52, 47.11)	12.98(−8.76, 38.82)
		GA	−5.13(−15.60, 4.60)	9.09(−17.82, 40.35)	−16.07(−71.77, 27.38)	8.87(−14.91, 36.21)	28.27(2.74, 60.16)
		AA	−3.98(−21.77, 12.75)	−13.09(−62.91, 27.51)	−57.30(−204.04, 23.00)	−3.56(−75.24, 63.39)	33.64(−28.27, 128.87)
		GA/ AA	−2.84(−10.85, 4.81)	−0.20(−21.65, 21.17)	−32.18(−80.40, 3.15)	4.60(−16.18, 27.12)	28.15(5.44, 55.74)
	rs763110						
		Interaction	0.59	0.43	0.28	0.91	0.38
		Stratified					
		CC	−5.87(−13.20, 1.01)	5.55(−16.77, 30.08)	−10.63(−52.50, 24.61)	4.71(−18.53, 29.82)	29.05(7.04, 55.58)
		CT	−4.08(−15.14, 6.18)	−5.13(−34.31, 21.53)	−52.50(−128.42, −1.82)	12.30(−10.96, 39.93)	21.05(−5.87, 55.12)
		TT	/	/	/	/	/
		CT/ TT	−2.74(−11.29, 5.55)	−5.13(−29.30, 17.00)	−52.50(−114.04, −8.65)	9.64(−10.63, 32.98)	16.88(−4.50, 42.90)
	rs12108497						
		Interaction	0.83	0.29	0.19	0.78	0.05
		Stratified					
		TT	−4.39(−13.31, 4.08)	14.45(−12.41, 47.11)	−3.25(−49.78, 40.49)	11.63(−14.91, 43.33)	7.90(−14.22, 32.84)
		TC	−3.36(−11.74, 4.71)	−12.19(−35.80, 7.90)	−69.38(−133.50, −22.88)	4.92(−15.37, 27.00)	30.73(5.76, 61.61)
		CC	−15.26(−46.67, 10.52)	−34.04(−253.60, 96.80)	−84.41(−569.93, 96.80)	−21.29(−262.92, 146.45)	166.45(−21.41, 761.93)
		TC/ CC	−4.6(−11.96, 2.33)	−7.14(−28.92, 12.41)	−55.74(−108.55, −16.30)	8.11(−11.52, 30.21)	36.75(12.64, 66.03)
1-OHP							
	rs2234767						
		Interaction	0.10	0.15	0.37	0.15	0.49
		Stratified					

	GG	−5.97(−12.98, 0.60)	14.11(−11.18, 44.77)	7.47(−33.24, 54.03)	18.41(−7.14, 50.08)	13.54(−7.79, 39.10)
	GA	−5.13(−13.20, 2.43)	20.20(−1.01, 45.79)	16.65(−16.65, 58.57)	19.48(0.60, 41.91)	0.70(−19.12, 20.68)
	AA	8.65(−6.82, 26.24)	−41.20(−93.67, −3.05)	−55.12(−193.88, 22.14)	−18.89(−96.6, 38.96)	17.23(−45.64, 100.17)
	GA/ AA	−1.92(−8.44, 4.39)	9.20(−7.47, 28.02)	4.29(−24.61, 35.53)	10.30(−6.50, 29.43)	0.80(−17.23, 19.12)
rs763110						
	Interaction	0.41	0.18	0.10	0.42	0.89
	Stratified					
	CC	−5.34(−11.96, 1.01)	15.14(−4.92, 39.10)	16.53(−14.91, 56.21)	13.31(−7.36, 37.85)	6.82(−11.85, 27.51)
	CT	−3.05(−11.29, 4.71)	4.50(−15.26, 25.86)	−2.12(−40.49, 34.58)	8.44(−8.98, 28.15)	8.11(−12.08, 30.87)
	TT	/	/	/	/	/
	CT/ TT	−2.43(−9.75, 4.71)	2.94(−16.18, 23.12)	−7.57(−46.23, 26.36)	7.90(−9.42, 27.51)	8.87(−9.53, 29.82)
rs12108497						
	Interaction	0.13	0.08	0.21	0.12	0.40
	Stratified					
	TT	−1.31(−7.79, 5.02)	2.12(−18.18, 23.37)	−7.90(−42.19, 22.14)	7.47(−11.96, 29.43)	1.92(−14.57, 19.01)
	TC	−6.18(−16.3, 3.05)	11.07(−12.52, 38.68)	14.80(−30.47, 71.77)	6.82(−16.88, 33.24)	10.85(−16.77, 43.48)
	CC	−11.74(−33.91, 7.14)	76.47(5.34, 195.65)	30.60(−117.93, 271.73)	102.38(27.76, 220.91)	11.85(−182.64, 253.60)
	TC/ CC	−8.00(−16.42, −0.10)	26.62(3.46, 55.12)	31.78(−6.82, 85.34)	22.14(−0.50, 50.08)	10.96(−13.54, 39.79)
∑OHNa						
rs2234767						
	Interaction	0.56	0.98	0.61	0.78	0.09
	Stratified					
	GG	−1.21(−4.81, 2.33)	−3.98(−18.18, 9.31)	−16.77(−40.92, 3.36)	0.50(−13.20, 14.34)	11.40(0.20, 23.86)
	GA	2.33(−2.12, 6.93)	−6.50(−19.72, 5.55)	−15.60(−38.40, 3.56)	−2.33(−13.66, 8.55)	−2.84(−14.57, 8.22)
	AA	5.65(−2.02, 13.88)	−9.97(−31.52, 8.87)	−0.30(−42.19, 41.34)	−19.24(−52.96, 7.47)	−19.24(−55.58, 9.31)
	GA/ AA	3.46(−0.20, 7.25)	−8.65(−19.24, 1.01)	−14.22(−32.98, 1.92)	−6.29(−16.88, 3.36)	−4.71(−15.49, 5.23)

rs763110						
	Interaction	0.32	0.96	0.88	0.83	0.66
	Stratified					
	CC	2.43(−0.90, 5.87)	−7.47(−19.01, 3.05)	−15.03(−34.45, 1.71)	−3.56(−15.14, 7.47)	3.46(−6.40, 13.88)
	CT	0.20(−4.81, 5.23)	−5.34(−18.53, 6.82)	−11.96(−37.03, 9.31)	−4.39(−16.18, 6.72)	−0.70(−13.88, 12.30)
	TT	/	/	/	/	/
	CT/ TT	−0.20(−4.60, 4.08)	−4.50(−16.42, 6.61)	−16.18(−39.38, 3.36)	−2.63(−13.66, 7.79)	1.82(−9.31, 13.31)
rs12108497						
	Interaction	0.09	0.07	0.20	0.21	0.26
	Stratified					
	TT	4.71(0.80, 8.65)	−15.49(−29.43, −3.05)	−25.23(−47.85, −5.97)	−9.64(−23.12, 2.33)	−4.08(−14.68, 5.87)
	TC	−0.60(−4.71, 3.46)	−3.98(−14.45, 5.97)	−14.22(−35.53, 3.87)	−2.33(−12.64, 7.57)	7.25(−4.08, 19.84)
	CC	−13.20(−38.68, 8.33)	61.77(−26.24, 230.69)	66.36(−80.76, 400.28)	68.37(−30.60, 270.62)	37.85(−155.49, 385.01)
	TC/ CC	−0.80(−4.39, 2.74)	−1.61(−11.96, 8.33)	−10.63(−29.43, 5.87)	−0.20(−10.41, 9.86)	6.72(−4.19, 18.53)
∑OHFlu						
rs2234767						
	Interaction	0.62	0.40	0.72	0.33	0.58
	Stratified					
	GG	−4.71(−9.86, 0.20)	10.96(−7.68, 32.58)	−2.22(−33.78, 28.02)	16.30(−2.53, 38.68)	−0.40(−17.12, 16.07)
	GA	−5.97(−13.20, 0.90)	8.44(−10.19, 29.69)	4.29(−26.87, 37.99)	1.11(−16.07, 18.65)	9.97(−6.93, 29.30)
	AA	1.82(−10.63, 14.57)	−9.31(−43.48, 20.08)	−20.68(−100.77, 37.99)	−6.29(−56.99, 38.96)	7.90(−40.07, 62.91)
	GA/ AA	−2.94(−8.76, 2.63)	1.92(−13.20, 17.59)	−5.65(−33.38, 19.36)	0.50(−14.91, 15.95)	9.97(−5.34, 27.38)
rs763110						
	Interaction	0.35	0.06	0.11	0.14	0.67
	Stratified					
	CC	−4.29(−9.42, 0.60)	11.96(−3.67, 29.82)	5.97(−18.89, 33.51)	10.52(−5.55, 28.92)	7.79(−6.61, 23.86)

	CT	−1.92(−9.75, 5.65)	−5.34(−25.99, 13.54)	−22.63(−66.03, 10.30)	0.30(−17.23, 18.06)	7.68(−11.63, 29.43)
	TT	/	/	/	/	/
	CT/ TT	−1.21(−7.47, 4.92)	−6.61(−24.11, 9.20)	−26.74(−63.56, 1.92)	−1.51(−17.23, 13.66)	5.97(−9.64, 23.12)
rs12108497						
	Interaction	0.44	0.32	0.22	0.48	0.02
	Stratified					
	TT	−1.11(−6.82, 4.50)	10.52(−6.93, 30.60)	5.02(−21.90, 34.58)	10.41(−6.82, 30.21)	−7.90(−23.86, 6.40)
	TC	−4.39(−10.96, 1.92)	−4.81(−22.02, 11.18)	−26.74(−66.03, 3.36)	0.20(−16.18, 16.53)	21.17(2.22, 43.62)
	CC	15.84(−25.11, 68.03)	−109.59(−626.45, 65.20)	−253.60(−1647.90, 39.65)	−105.44(−745.70, 100.17)	−11.07(−879.63, 694.86)
	TC/ CC	−4.71(−10.63, 0.80)	−2.74(−19.36, 13.20)	−22.75(−56.83, 4.19)	1.51(−14.68, 18.06)	20.56(2.63, 41.62)
∑OHph						
rs2234767						
	Interaction	0.79	0.80	0.43	0.65	0.87
	Stratified					
	GG	−0.90(−2.84, 1.01)	−0.20(−7.47, 7.04)	−7.68(−19.36, 2.84)	2.63(−4.50, 10.08)	2.12(−3.87, 8.44)
	GA	−2.12(−4.60, 0.30)	7.57(1.01, 14.45)	5.34(−4.92, 16.53)	5.55(−0.20, 11.74)	3.25(−2.74, 9.53)
	AA	−0.60(−4.71, 3.46)	−3.98(−13.88, 5.44)	−8.11(−28.27, 9.75)	−3.05(−17.59, 10.63)	5.65(−8.55, 21.29)
	GA/ AA	−1.31(−3.36, 0.60)	3.67(−1.41, 8.98)	1.01(−7.47, 9.64)	2.84(−2.22, 8.11)	3.36(−1.92, 8.76)
rs763110						
	Interaction	0.35	0.24	0.11	0.66	0.79
	Stratified					
	CC	−1.71(−3.67, 0.10)	2.84(−2.94, 8.98)	1.41(−7.90, 10.85)	1.82(−4.29, 8.00)	3.56(−1.82, 9.20)
	CT	−0.50(−3.25, 2.12)	−0.30(−6.93, 6.29)	−7.47(−19.72, 3.67)	2.53(−3.36, 8.65)	2.84(−3.87, 9.86)
	TT	/	/	/	/	/
	CT/ TT	−0.40(−2.53, 1.71)	−0.60(−6.18, 5.02)	−9.09(−19.36, 0.30)	2.12(−3.05, 7.36)	2.74(−2.63, 8.33)
rs12108497						

	Interaction	0.86	0.40	0.17	0.89	0.06
	Stratified					
	TT	−1.11(−3.25, 0.90)	4.19(−2.12, 10.96)	1.92(−7.68, 11.74)	3.46(−2.84, 10.08)	−0.30(−5.65, 5.02)
	TC	−1.11(−3.46, 1.21)	−1.82(−7.68, 3.87)	−11.40(−22.88, −1.01)	1.01(−4.60, 6.82)	6.40(−0.10, 13.31)
	CC	−0.90(−17.7, 15.49)	19.96(−41.06, 102.99)	−7.47(−134.43, 103.20)	31.13(−31.92, 126.82)	22.38(−84.41, 176.49)
	TC/ CC	−1.31(−3.46, 0.70)	−0.50(−6.18, 5.23)	−9.53(−19.72, −0.10)	2.33(−3.36, 8.22)	7.04(0.90, 13.66)
∑OHAH						
rs2234767						
	Interaction	0.73	0.85	0.69	0.48	0.66
	Stratified					
	GG	−0.70(−1.82, 0.30)	0.40(−3.46, 4.29)	−3.56(−9.64, 2.12)	2.02(−1.92, 5.97)	1.92(−1.31, 5.23)
	GA	−0.80(−2.22, 0.50)	2.63(−1.01, 6.40)	0.90(−4.81, 6.82)	2.12(−1.11, 5.44)	1.11(−2.22, 4.60)
	AA	0.60(−1.82, 3.05)	−3.56(−9.20, 1.92)	−4.81(−16.18, 5.76)	−3.46(−11.85, 4.50)	1.11(−7.57, 9.97)
	GA/ AA	−0.30(−1.41, 0.80)	0.80(−2.12, 3.77)	−1.01(−5.87, 3.77)	0.70(−2.22, 3.67)	1.11(−1.92, 4.08)
rs763110						
	Interaction	0.59	0.24	0.17	0.47	0.88
	Stratified					
	CC	−0.70(−1.71, 0.40)	1.11(−2.12, 4.50)	−0.20(−5.44, 4.92)	1.11(−2.33, 4.60)	2.02(−1.01, 5.23)
	CT	−0.30(−1.71, 1.11)	−0.50(−3.98, 2.94)	−3.87(−9.97, 2.02)	0.60(−2.43, 3.77)	1.31(−2.22, 4.92)
	TT	/	/	/	/	/
	CT/ TT	−0.30(−1.41, 0.90)	−0.70(−3.77, 2.33)	−5.02(−10.41, 0.10)	0.60(−2.22, 3.46)	1.41(−1.51, 4.50)
rs12108497						
	Interaction	0.28	0.86	0.64	0.67	0.04
	Stratified					
	TT	0(−1.21, 1.11)	0.50(−3.05, 4.08)	−1.41(−6.82, 3.77)	0.90(−2.53, 4.50)	−0.70(−3.67, 2.22)
	TC	−0.70(−2.02, 0.60)	−0.90(−4.08, 2.22)	−5.55(−11.40, 0.10)	0.20(−2.94, 3.36)	3.67(0.10, 7.36)

	CC	−2.43(−10.19, 4.92)	15.26(−10.08, 46.23)	2.33(−44.20, 51.13)	20.56(−5.55, 53.27)	9.86(−36.75, 65.04)
	TC/ CC	−0.90(−2.02, 0.30)	0.10(−3.05, 3.25)	−4.29(−9.75, 0.90)	1.21(−1.92, 4.50)	3.98(0.50, 7.57)

[a] Multiple linear regression analyses were used, correcting for age, BMI, pregnacy, income, education, duration of abstinence, smoking status and alcohol consumption.

[b] / not analysed due to insufficient sample size.

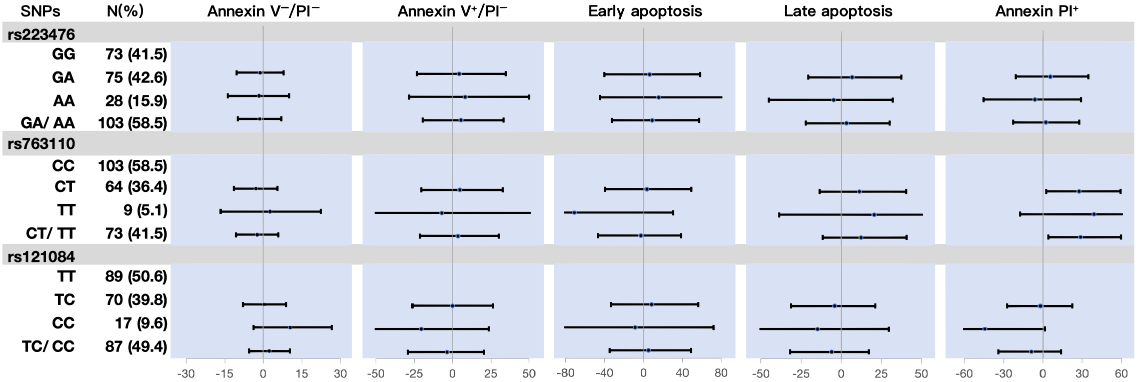

Figure S1 Percentage change for spermatozoa apoptosis with Fas, FasL and caspase-3 genotypes. Multiple linear regression analyses were used, correcting for age, BMI, pregnacy, income, education, duration of abstinence, smoking status and alcohol consumption. The wild-type homozygote was designated as the reference group; Fas rs2234767 GG, FasL rs763110 CC, and caspase-3 rs12108497 TT are wild-type homozygote; Fas rs2234767 GA, FasL rs763110 CT, and caspase-3 rs12108497 TC are heterozygote; Fas rs2234767 AA, FasL rs763110 TT, and caspase-3 rs12108497 CC are Variant homozygote; Fas rs2234767 GA/AA, FasL rs763110 CT/TT, and caspase-3 rs12108497 TC/CC are combined genotype.
